# Declining venom immunotherapy: patient characteristics and clinical outcomes

**DOI:** 10.1186/s13223-026-01046-w

**Published:** 2026-06-15

**Authors:** Simon Ueberschaar, Axel Trautmann, Johanna Stoevesandt

**Affiliations:** 1 Department of Dermatology, Venereology, and Allergology, University Hospital Würzburg, Josef-Schneider-Straße 2, 97080 Würzburg, Germany; 2 Dermatology Practice Ueberschaar, Sonthofen, Germany

**Keywords:** Anaphylaxis, Honey bee, Natural course, Risk factor, Shared decision making, Treatment adherence, *Vespula*

## Abstract

**Background:**

A largely unknown proportion of *Hymenoptera* venom-allergic patients do not undergo venom immunotherapy (VIT) despite positive allergy testing and counselling. We aimed to identify factors associated with the refusal of VIT, and evaluate the natural course of venom allergy in untreated individuals.

**Methods:**

Out of 1163 candidates for VIT, 271 (23.3%) declined or postponed treatment for at least 12 months. Complete data from 166 of these patients, who were interviewed and counselled during routine follow-up, were available for retrospective evaluation.

**Results:**

Patients declining VIT were significantly more likely to be female (*P* = 0.012) and had a lower grade of index sting-induced anaphylaxis (*P* < 0.001) compared to those who accepted treatment. Main reasons for deciding against VIT were that it was considered too time-consuming (39.8%) or perceived as unnecessary (24.1%). A minority of patients (6.6%) declined VIT due to fear of side effects; 7.2% perceived the VIT-associated risk as high to extremely high. Most (69.9%) reported routinely carrying an epinephrine autoinjector. The re-sting rate was 39.2% over a median follow-up of 5 years. Among 65 re-exposed patients, 13 (20%) reported an anaphylactic sting-reaction, with only two cases classified as severe. In no case did a severe relapse occur following a mild index sting reaction.

**Conclusion:**

In venom-allergic patients hesitant about VIT, treatment choices should be guided by a shared decision-making process considering risk factors for severe anaphylaxis and the degree of sting exposure, together with the patient’s personal needs and preferences.

**Supplementary Information:**

The online version contains supplementary material available at 10.1186/s13223-026-01046-w.

## Background

An estimated 60–90% of individuals sustain at least one sting from *Hymenoptera* species—most commonly from bees or vespids—at some point during their lifetime [1–3]. Up to 7.5% of adults stung develop an anaphylactic reaction [1], making *Hymenoptera* venom allergy a leading cause of anaphylaxis in many countries worldwide [4]. Whereas food allergy is the predominant trigger of anaphylaxis in toddlers, insect venom allergy becomes increasingly important in older children and adolescents [5]. Venom immunotherapy (VIT) is an effective treatment for preventing future anaphylactic sting reactions in individuals with *Hymenoptera* venom allergy [6], and current international treatment guidelines recommend VIT for all patients with a history of moderate-to-severe sting-induced anaphylaxis [1]. However, a substantial but largely unknown proportion of eligible patients do not receive VIT [7–9]. Two main factors may explain this apparent gap in care: (i) Due to a lack of referral for allergy evaluation and counselling, patients remain unaware of VIT as the treatment of choice [8, 9] (ii) Despite adequate counselling in accordance with guidelines, patients may decide against VIT for various reasons, such as concerns about the burden or complexity of the treatment, or fear of potential side effects. While the first group might be reached through targeted training of medical personnel and broad public awareness campaigns, our single-centre retrospective observational cohort study focuses on the latter: patients who have made an informed decision to decline VIT despite an allergist’s recommendation. The following points are addressed:

(i) What proportion of patients opts against the recommended VIT despite an allergist’s advice?
(ii) Do the clinical and demographic baseline characteristics of patients who decline VIT differ from those who adhere to the guideline-recommended therapy?
(iii) What factors contribute to patients declining VIT?
(iv) What is the natural course of *Hymenoptera* venom allergy in untreated patients?

## Methods

### Patient cohort

The Allergy Centre Mainfranken is a tertiary referral centre specialising in the management of patients with insect venom allergy. It receives referrals from within a radius of approximately 100 km. This retrospective single centre observational study includes 1163 consecutive patients who were diagnosed with *Hymenoptera* venom allergy and met the criteria for VIT at our clinic over a 10-year period (2011–2021). In accordance with national and international guidelines applicable during the study period, *Hymenoptera* venom allergy was diagnosed in patients with a history of sting-induced anaphylaxis, provided that sensitisation was confirmed by intradermal testing, serological detection of venom-specific IgE, or basophil activation testing [10–13]. VIT was recommended for patients with a history of moderate-to-severe systemic sting reactions, and—based on individual assessment—for those with mild anaphylaxis if risk factors for severe reactions were present [14, 15], such as elevated serum tryptase levels, underlying mastocytosis, advanced age, or increased exposure. Each patient received detailed medical counselling regarding VIT and was provided with an allergy ID card, a printed emergency action plan, and an emergency kit for self-administration including an epinephrine autoinjector.

### Follow-up of patients declining VIT

Routine medical care at our allergy centre includes follow-up of patients who decline the recommended VIT. Patients are classified as “untreated” or “having declined treatment” if, within 12 months following allergy testing and counselling, they have neither initiated VIT nor scheduled an appointment for treatment initiation. Untreated patients are offered a follow-up counselling session in person or alternatively by phone. During the follow-up consultation, patients are re-advised to undergo VIT. As part of our internal quality management, the reasons for refusing VIT, along with the individual perception of risks associated with VIT and potential future sting reactions, are explored using a standardised questionnaire (Supplementary Material 1). Additionally, patients are inquired about the occurrence and outcome of field stings from bees or vespids, and whether they carry an emergency kit. As an integral part of follow-up counselling, patients receive refresher training on the correct use of the epinephrine autoinjector.

### Data management

The local institutional review board approved the retrospective review and publication of anonymised clinical data. Complete digital and paper medical records from 1163 consecutive patients diagnosed with *Hymenoptera* venom allergy, who were advised to undergo VIT, were available for retrospective evaluation. Clinical data included patients’ sex, age, comorbidities, and the time and severity of the index sting reaction. Laboratory data comprised baseline serum tryptase levels (ImmunoCAP™, Thermo Fisher Scientific, Freiburg, Germany). The aforementioned questionnaire, included in the patient’s paper medical record, was reviewed during data collection. If no questionnaire was available, the respective case was classified as “lost to follow-up”. All patient-related data were pseudonymised and stored in a password-protected file, accessible only to the authors of this study. All data were fully anonymised prior to analysis.

Data from patients who followed the VIT recommendation have been published independently in other studies from our group [16, 17].

### Classification of sting-induced reactions

Anaphylaxis was classified as mild, moderate, or severe according to a modified version of the grading system proposed by Muraro et al. [18, 19]: (1) mild, with symptoms largely limited to skin and/or mucous membranes; (2) moderate, with objective extracutaneous involvement of cardiovascular, respiratory, gastrointestinal, or neurological systems; and (3) severe, characterised by serious systemic symptoms such as oxygen desaturation, severe hypotension, loss of consciousness, or cardiac arrest. Large local reactions were defined as exceeding 10 cm in diameter and lasting more than 24 h.

### Statistical analyses

Statistical analyses were performed using SPSS version 29 for Windows (IBM SPSS, Armonk, NY). Interval-scaled data are presented as medians with the corresponding range. Ordinal and categorical data are reported as absolute and relative frequencies. The Mann-Whitney U test was applied to compare two independent groups for interval-scaled data. The Chi-square test, or Fisher’s exact test when applicable, was used to analyse categorical data. All tests were two-tailed, with a *P* value of < 0.05 considered statistically significant.

## Results

### Patient cohort

Of 1163 consecutive patients with confirmed bee and/or vespid venom allergy who were advised to start VIT, 892 presented for initiation of treatment. The remaining 271 (23.3%) refused or postponed VIT for at least 12 months. Clinical baseline data on treated and untreated patients are depicted in Table 1. The total cohort had a median age of 45 (range 4–84) years at the time of the index sting. No significant difference in age was observed between patients who received VIT and those who declined treatment. Similarly, there were no statistically significant differences between the two groups in terms of diagnosis (*Vespula* venom allergy, bee venom allergy, or double allergy), or history of cardiovascular comorbidities. Male patients predominated in the total group and the group receiving VIT (males 55.6%, females 44.4%), whereas the group that declined treatment included a significantly higher proportion of females (53.1%, *P* = 0.012). The two groups also differed significantly with respect to the severity of the index sting-induced anaphylactic reaction, with patients receiving VIT having a significantly higher frequency of moderate-to-severe reactions (*P* < 0.001). In the group that declined therapy, 28.4% had experienced only mild cutaneous symptoms (urticaria, angioedema), indicating a relative indication for VIT. However, 55.4% reported moderate, and 16.2% severe anaphylaxis.

**Table 1:** Patient cohort

		Untreated		(%)	VIT		(%)		Total		*P*
Number of patients		271			892				1163		
Age at time of index sting, Median (years) (range)		43.8 (6–83)			46 (4–84)				45 (4–84)		0.81
Sex											
Male		127		(46.9)	496		(55.6)		623	(53.6)	**0.012**
Female		144		(53.1)	396		(44.4)		540	(46.4)	
Severity of anaphylaxis											
Mild		77		(28.4)	122		(13.7)		199	(17.1)	**< 0.001**
Moderate		150		(55.4)	546		(61.2)		696	(59.8)	
Severe		44		(16.2)	224		(25.1)		268	(23.0)	
Diagnosis											
*Vespula* venom allergy		225		(83.0)	720		(80.7)		945	(81.3)	0.57
Bee venom allergy		41		(15.1)	147		(16.5)		188	(16.2)	
Double allergy		5		(1.8)	25		(2.8)		30	(2.6)	
Concomitant cardiovascular disease											
Yes		69		(25.5)	245		(27.5)		314	(27.0)	0.53
No		202		(74.5)	647		(72.5)		849	(73.0)	
Median tryptase concentration, µg/L (range)		4.2 (≤ 1.0-28.6)			4.3 (≤ 1.0-148.0)				4.3 (≤ 1.0-148.0)		0.88

### Self-assessment of patients refusing VIT

**Fig. 1:**
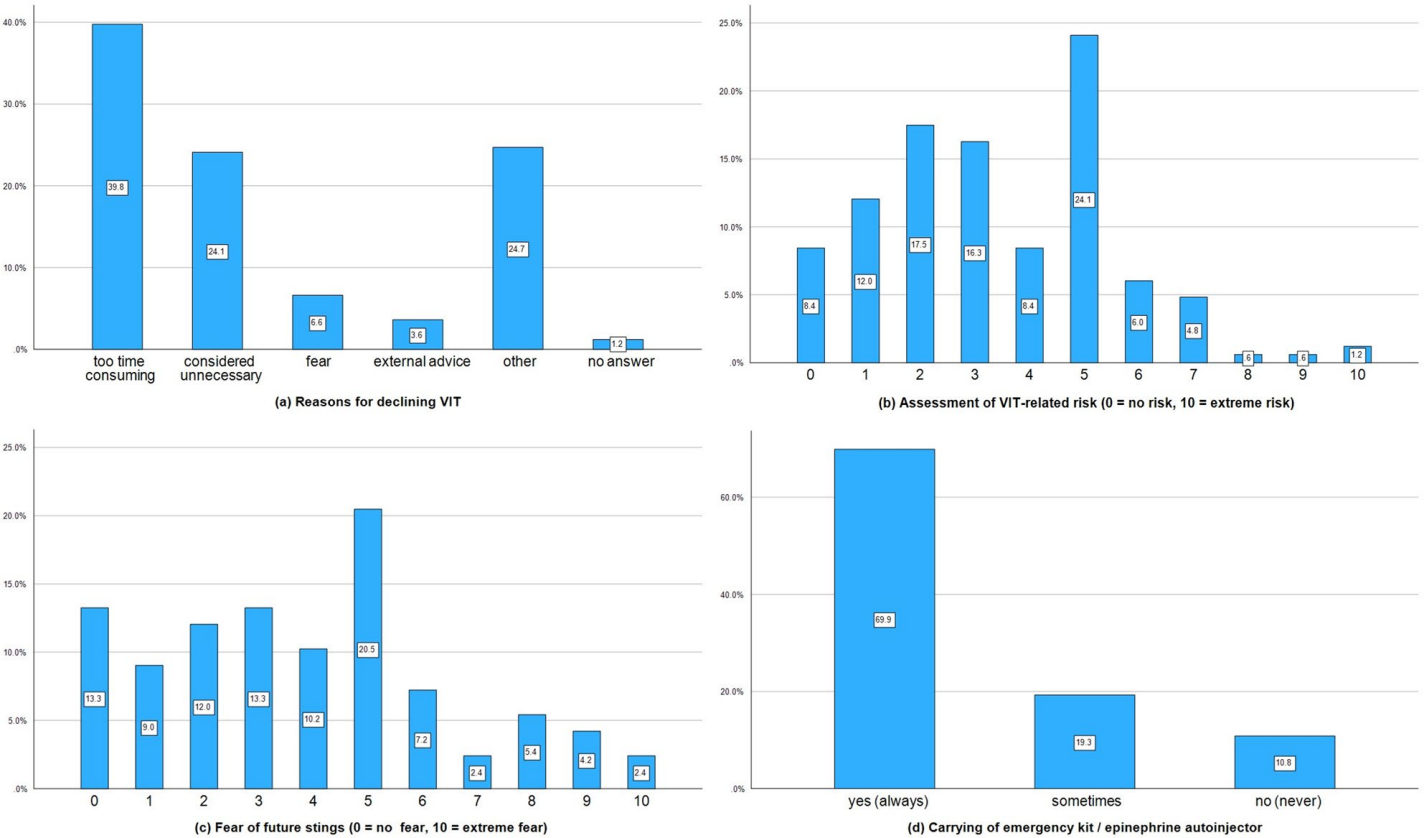
Self-assessment of 166 patients declining VIT

Of the 271 patients who did not undergo VIT, 105 (38.7%) were lost to follow-up. The remaining 166 (61.3%) accepted the offer of one or several follow-up consultations, either in person or via telephone. Patients’ self-assessments during the follow-up counselling are summarised in Fig. 1a–d. The most frequently reported reason for deciding against VIT was that it was considered too burdensome or time-consuming (39.8%), a further 24.1% of patients did not consider VIT necessary. Only a minority of patients (6.6%) did not start VIT due to fear of side effects (Fig. 1a). The majority of patients perceived VIT to be associated with a low to moderate risk (Fig. 1b) and rated their fear of future stings as at most moderate (0–5 on a 10-point numerical rating scale) (Fig. 1c). The majority of patients (69.9%) reported that they routinely carried an emergency kit containing an epinephrine autoinjector (Fig. 1d).

### Field sting exposure and outcomes

**Fig. 2:**
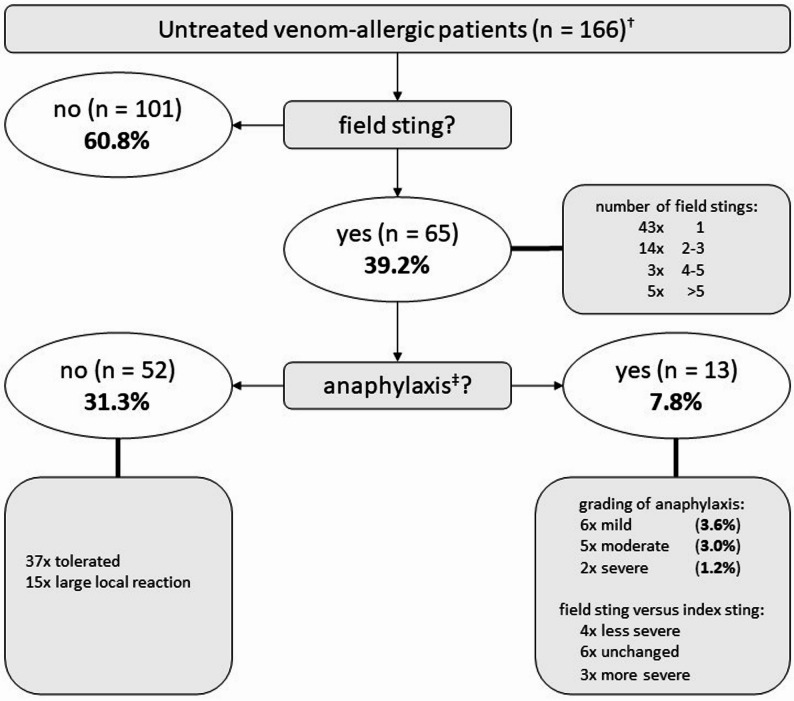
Field sting exposure and outcomes in untreated venom allergic patients. ^†^Percentages given refer to the total number of patients available for follow-up. ^‡^For patients with multiple field stings, the most severe (or, in case of equal severity, the most recent) reaction was evaluated

The median time interval from the initial diagnosis/counselling to the last documented patient contact was 5 (range: 1–11) years. During this follow-up period, 65 (39.2%) of the 166 patients available for follow-up evaluation had experienced one or several stings by the relevant *Hymenoptera* species (Fig. 2). Fifty-two patients—representing 80% of those re-exposed and 31.3% of all patients available for follow-up—tolerated field stings or developed only a large local reaction. Additional information on the remaining 13 patients (20% of those re-exposed, 7.8% of those available for follow-up) who experienced re-sting-induced anaphylaxis is provided in Table 2. All patients who developed re-sting reactions had initially been diagnosed with *Vespula* venom allergy. One female patient experienced a grade 2 reaction to a bee sting two years after her initial reaction to a *Vespula* sting; no sensitisation to bee venom had been detected in the initial allergy testing. All other patients reacted to vespid field stings. Six re-sting reactions were classified as mild, 5 as moderate, and 2 as severe (corresponding to 3.1% of re-exposed patients and 1.2% of those available for follow-up). In 3 cases, the field sting-induced anaphylaxis was more severe, in 4 cases less severe, and in the remaining 6 cases unchanged compared to the index sting. In no case did a severe (grade 3) relapse reaction occur following an initially mild (grade 1) reaction.

**Table 2:** Characteristics of 13 patients^†^ reacting to field stings^‡^

Patient	Age	Sex	Grade of index sting reaction	Field stings (*n*)	Systemic reactions to field stings (*n*)	Time interval^‡,§^	Insect causing field sting reaction^‡^	Grade of field sting reaction^‡,¶^	Use of epinephrine autoinjector after field sting^‡^
#1	22	f	3	1	1	13	*Vespula*	2 (↓)	no
#2	35	f	1	1	1	4	*Vespula*	2 (↑)	no
#3	36	m	2	1	1	6	*Vespula*	1 (↓)	no
#4	43	f	2	3	3	10	*Vespula*	1 (↓)	yes
#5	43	m	2	1	1	5	*Vespula*	2 (-)	yes
#6	46	f	2	1	1	< 1	*Vespula*	1 (↓)	no
#7	47	f	1	1	1	7	*Vespula*	2 (↑)	no
#8	51	f	2	1	1	2	honey bee	2 (-)	no
#9	55	f	2	1	1	9	*Vespula*	3 (↑)	yes
#10	57	f	1	3	1	8	*Vespula*	1 (-)	no
#11	62	m	1	7	7	6	*Vespula*	1 (-)	no
#12	67	f	1	1	1	< 1	*Vespula*	1 (-)	no
#13	77	f	3	2	2	5	*Vespula*	3 (-)	no

^†^All patients had an initial diagnosis of *Vespula venom* allergy; ^‡^for patients with several field stings, the most severe reaction was evaluated; if there were several reactions of equal severity, the most recent one was evaluated; ^§^time from index sting to field sting reaction (years); ^¶^severity of field sting reaction in comparison to index sting: more severe (↑), less severe (↓), same severity (-)

## Discussion

A substantial proportion of patients with a history of anaphylaxis to bee or vespid stings do not receive VIT. This is often attributed to insufficient counselling or a lack of awareness regarding VIT as the treatment of choice [8, 9]. Our study, however, focuses on a specific subgroup of patients: those who decline VIT despite appropriate diagnostic evaluation and comprehensive counselling. In our cohort, 271 of 1163 patients (23.3%) did not initiate VIT contrary to medical advice. Interestingly, patients who declined VIT were significantly more likely to be female compared to those who adhered to the treatment recommendation (*P* = 0.012). Comparative data from other medical fields—particularly in the treatment of cardiovascular diseases—also indicate that women, compared to men, tend to exhibit a lower adherence to medical treatment recommendations [20, 21]. The underlying causes appear to be multifactorial, involving psychosocial and economic factors. Evidence regarding gender-specific adherence to allergen immunotherapy has been inconclusive so far [22, 23], whereas one study found that male patients indicated for subcutaneous immunotherapy with inhalant allergens or insect venoms were more likely to achieve the recommended maintenance dose compared to females [24].

Understanding the reasons for declining VIT may enable the implementation of targeted interventions aimed at improving treatment adherence. Economic reasons were not assessed in our cohort, as the costs of VIT are fully reimbursed in Germany. In countries where this is not the case, therapy costs have been identified as a major obstacle to treatment [25]. In our study, the main reason for deciding against VIT was the perception that it was too time-consuming (39.8%, Fig. 1a). It is particularly the build-up phase of VIT—which in our centre is routinely carried out as a 3-day inpatient rush protocol [26]—that may be perceived as difficult to reconcile with professional or family obligations. To address this issue, patients may be given the option to choose an outpatient up-dosing protocol. In addition to the conventional multi-week outpatient schedule, clustered protocols [27], a 7-week abbreviated conventional protocol [28, 29], or ultra-rush protocols including a 10-step, single day protocol [30] are considered viable options.

The second most common reason for opting against VIT was the belief that it was unnecessary (24.1%). In a minority of cases (3.6%), the decision to reject treatment was based on external advice, usually provided by general practitioners or other physicians. Only a relatively small percentage of patients (6.6%) explicitly cited fear or anxiety as the reason for rejecting VIT. Beyond providing objective, informative counselling and, if necessary, facilitating access to competent external advice, it is recommended to specifically explore patients’ treatment expectations and actively enhance benefit expectations whenever possible [31]. Treatment-related adverse effects should be openly discussed, with information on their frequency positively framed [31], for example: “more than 90% of patients tolerate VIT without systemic reactions”.

In our cohort, 39.2% of patients experienced one or more re-stings by the relevant insect within a median follow-up of five years (Fig. 2). Systemic sting reactions occurred in only 20% of re-exposed individuals, corresponding to an overall rate of 7.8% for re-sting-induced anaphylaxis. Assuming a 5% reaction rate upon re-sting in individuals undergoing VIT [1], the resulting number needed to treat (NNT) is 17 to prevent one anaphylactic re-sting reaction.

The observed relapse rate is lower than anticipated based on previously published data. Historical studies reported a relapse rate of systemic anaphylactic sting reactions of up to 74% in adult insect venom-allergic patients [32], whereas lower rates—ranging from 13 to 32%, depending on the severity of the initial reaction—have been described for children [33]. In a 2012 Cochrane Database systematic review, an overall relapse rate of 39.8% was reported among 93 untreated venom-allergic patients who were accidentally or intentionally re-exposed to stings by the relevant insect [6]. The authors confirmed that VIT is effective in preventing further anaphylactic sting reactions, but due to the small number of fatal cases, they were unable to statistically demonstrate its effectiveness in preventing fatal outcomes [6]. Consistent with this observation, the risk of a fatal anaphylactic reaction to an insect sting—even in individuals with a known insect venom allergy—is considered so low that it adds only minimally to the overall mortality risk [34].

The relatively broad recommendation of VIT for all patients with a moderate-to-severe systemic sting reaction [1]—even though only a subset will be stung again, not all will react to a subsequent sting, and only a negligible number will experience a fatal reaction—is commonly justified by the anticipated improvement in patients’ quality of life [1, 6, 35–37]. Published studies assessing health-related quality of life in venom-allergic patients, however, only included individuals who were either awaiting VIT [35] or were randomised into one of two treatment groups (VIT versus epinephrine autoinjector) [36, 37]. It can be expected that patients who are sceptical of VIT, perceiving it as unnecessary, overly burdensome, or dangerous, are less likely to experience VIT-related improvements in their quality of life.

**Fig. 3:**
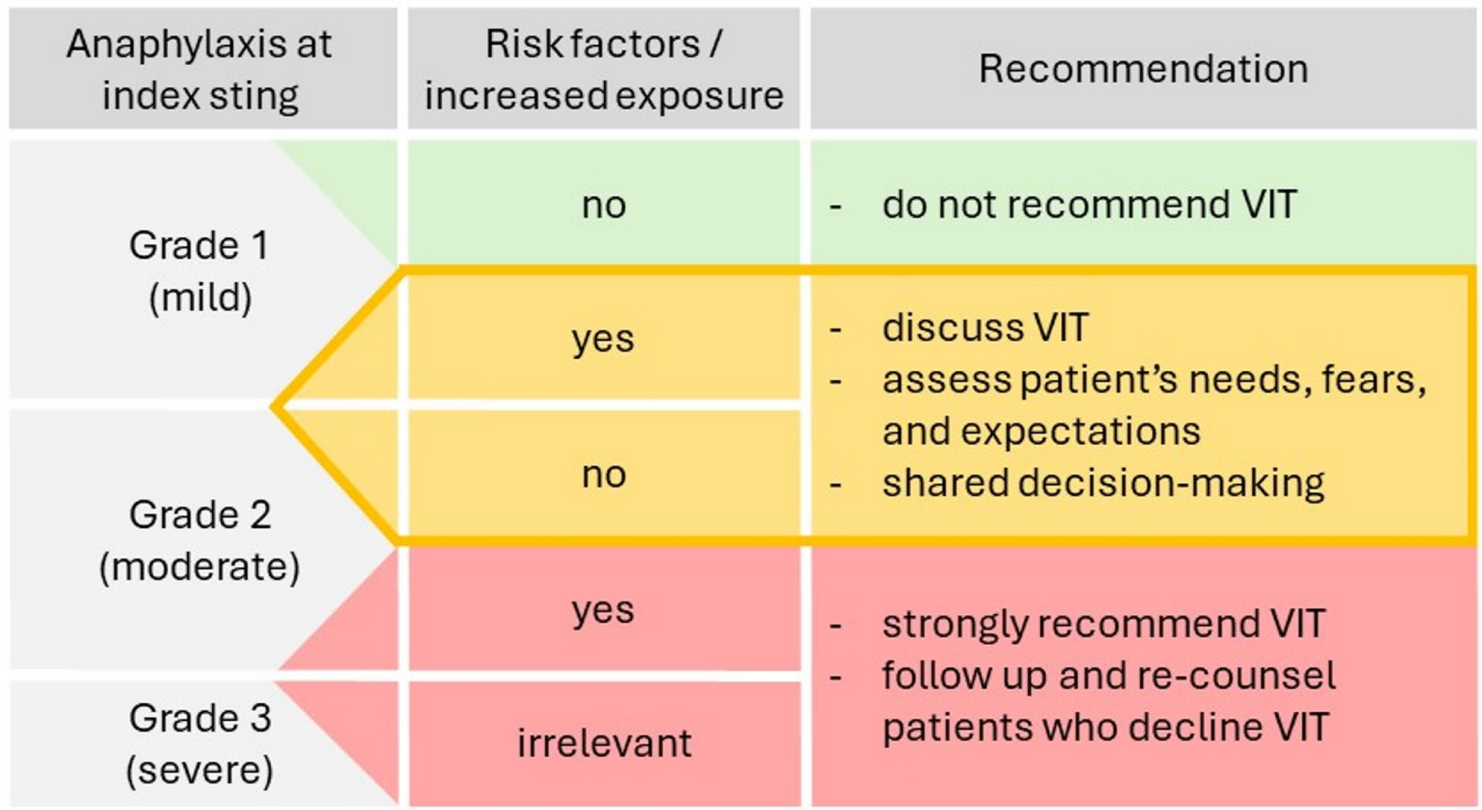
Individualised counselling and recommendation of VIT, taking into account anaphylaxis severity, risk factors/exposure, and the patient’s needs, fears, and expectations

Notably, patients who declined VIT exhibited significantly milder index-sting-induced anaphylaxis compared to those who followed the recommendation of treatment (*P* < 0.001, Table 1). This is probably due to the fact that the group of patients declining treatment included a larger proportion of individuals with a relative indication for VIT—that is, patients who had experienced only mild (grade 1) anaphylaxis following their index sting and who were recommended VIT because of coexisting risk factors or increased exposure to insect stings. Literature suggests a correlation between the severity of previous anaphylactic sting reactions and that of future reactions [32, 33]. Our findings align with this observation: although severity grades varied between the initial index reaction and subsequent relapse reactions, no patients progressed from an initially mild (grade 1) to a severe (grade 3) relapse reaction (Table 2). Our own data on the natural course of insect venom allergy, consistent with the aforementioned studies [6, 32–34], suggest that withholding VIT is justifiable when the informed patient explicitly opts against it. However, a careful evaluation of risk factors for severe sting reactions is necessary to identify patients who should be prioritised for VIT and clearly advised about the critical necessity of the treatment [14, 15]. In summary, our findings indicate that a strictly binary classification of “VIT recommended” versus “not recommended” does not adequately capture the complex medical and personal considerations involved in counselling patients with insect venom allergy. Instead, a nuanced approach should be adopted, establishing a flexible decision zone in which, following a thorough assessment of patients’ needs, fears, and expectations, a shared decision-making process can guide the choice for or against VIT (Fig. 3).

### Study limitations

The limitations of our study lie in its retrospective design, with data derived from routine patient care: (i) Patients who accepted the offer of re-counselling may have done so due to experiencing a re-sting and possibly a re-sting related anaphylactic reaction (selection bias); (ii) Patients may have responded in a way they believed was expected or desired by their treating allergist, for example, when asked about carrying the emergency kit (response bias) (iii) Only a relatively small number of re-sting reactions were reported, all of which occurred in *Vespula* venom-allergic patients. No conclusions can be drawn regarding the natural course of bee venom allergy. (iv) As is common with self-reported field-sting data, patients may have incorrectly identified the stinging insect.

## Conclusion

The observed low rates of re-sting reactions, and especially the rarity of severe anaphylaxis, indicate that a re-evaluation of the current criteria for initiating VIT is warranted. In venom-allergic patients hesitant about VIT, treatment choices should be guided by a shared decision-making process that carefully considers individual risk factors for severe anaphylaxis, sting exposure, and the patient’s personal needs and preferences.

## Supplementary Information

Below is the link to the electronic supplementary material.

Supplementary Material 1

## Data Availability

The datasets analysed during the study are not publicly available in order to protect patients’ privacy but are available from the corresponding author upon reasonable request.

## Abbreviations

VIT: Venom immunotherapy
