# Supplementary material for: Declining venom immunotherapy: patient characteristics and clinical outcomes
Source: Allergy Asthma Clin Immunol. 2026 Jun 15;22:38. doi: 10.1186/s13223-026-01046-w (PMC13270586; doi:10.1186/s13223-026-01046-w)
Supplement: Supplementary file 1 — Supplementary Material 1 [file 13223_2026_1046_MOESM1_ESM.docx]

Interview to be completed by the treating physician (English translation)

Follow-up counselling of patients who have not initiated venom immunotherapy (VIT)

Name: ________________________
Date of Birth: ________________________

# **1. Self-assessment /** quality management

## **1.1 Select the most important reason: Why did you decide against starting VIT?**

(1) I don’t know / I don’t want to say
(2) I consider VIT unnecessary
(3) I received external medical advice against VIT
(4) I am afraid of VIT / afraid of side effects
(5) I don’t have time / organisational reasons
(6) Other reasons: _____________________________________________________________

1.2 Please rate on a scale from 0 to 10: How would you rate the quality of counselling at our clinic?
0 (extremely poor) to 10 (extremely good): ______

1.3 Please rate on a scale from 0 to 10: How would you assess the risk of side effects during VIT?
0 (no risk) to 10 (extreme risk): ______

1.4 **Please rate on a scale from 0 to 10:** How strong is your fear of future stings by wasps or honeybees?
0 (no fear) to 10 (extreme fear): ______

1.5 **Please select the appropriate answer:** Do you carry your emergency kit / epinephrine injector with you?
(0) No, never
(1) Sometimes
(2) Yes, always

2. Field stings after allergy testing / counselling
2.1 How often have you been stung again?
🞎 No sting occurred (**→** **skip 2.2–2.6**)
🞎 Stings by wasps or hornets, number (estimate if necessary): ______
🞎 Stings by honeybees or bumblebees, number (estimate if necessary): ______
🞎 Stings by unidentified insects, number (estimate if necessary): _____

2.2 **Please select the appropriate answers regarding your most recent field sting:**

a) Which insect most likely caused your most recent field sting?

🞎 Wasp or hornet
🞎 Honeybee or bumblebee
🞎 Unknown / not identified

b) When did your most recent field sting occur?

Date (dd.mm.yyyy, estimate if necessary): ____________

2.3 **Please select the appropriate answer: How did you react to field stings?**
🞎 Only normal local sting reactions (**→** **skip 2.4–2.6**)
🞎 Only large local reactions (exceeding 10 cm, lasting over 24 hours) occurred (**→** **skip 2.4–2.6**)
🞎 I had one or several systemic reactions (anaphylaxis),

🞎 **→** provide number of systemic reactions to field stings (estimate if necessary): ____________

2.4 **Please select the appropriate answers regarding your most severe field sting reaction:**

a) Which insect most likely caused your most severe field sting reaction?

🞎 Wasp or hornet
🞎 Honeybee or bumblebee
🞎 Unknown / not identified

b) When did your most severe field sting reaction occur?

Date (dd.mm.yyyy, estimate if necessary): ____________

**2.5 Please circle the appropriate information regarding the most severe sting reaction**Symptoms: Urticaria/angioedema, respiratory, gastrointestinal, hypotension, unconsciousness

Use of epinephrine autoinjector? 🞎 Yes 🞎 No
Intervention: none, outpatient, emergency medical services, inpatient, intensive care

2.6 **Please select the appropriate answer:** Classification of the most severe field sting reaction
(1) Anaphylaxis Grade I (mild)
(2) Anaphylaxis Grade II (moderate)
(3) Anaphylaxis Grade III (severe)

3. Checklist
🞎 Counselling on VIT provided
🞎 Counselling on emergency kit provided
🞎 Emergency kit prescribed

Physician: ________________________
Date: ________________________
Signature: ________________________
